# Matters of Medical News

**Published:** 1856-07

**Authors:** 


*Matters of Medical News.*—Prof. Mutter has resigned the chair of Surgery in the Jefferson Medical College, of Philadelphia. Prof. S. D. Gross, of Louisville, succeeds him. The appointment is a strong one. The transfer of Prof. Flint to Buffalo, and Prof. Gross to Philadelphia, is a serious loss to the Louisville school, but we hope to see their places filled by similar men, and the great school of the west continue in all its former prosperity.

Dr. Hills, of the Medical Counsellor, (Ohio) has been appointed Superintendent of the Central Lunatic Asylum, of Ohio. Dr. H. is a man of much energy and character.

The will of Dr. John C. Warren is a curious document. All his property, even to the skeleton of the mastodon, is kept in the family. The only public bequest (we understand) is his own skeleton, which is to be prepared and placed in the museum of the Medical College, at Boston. The will is peremptory in its provisions.

Alexis St. Martin is figuring in the medical world again. Dr. Bunting, of Montreal, has him in charge in the eastern cities, and is to take him to Europe. So far as we can gather, no positive benefit to science has yet been reaped. The experiments of Beaumont are repeated in a superficial way, but we do not learn of anything new. In fact the case has measurably lost its interest since gastric fistulæ have been so freely established in the lower animals. It only remains to prove that the gastric juice of man and the dog is identical.

The "New England Female College" bill has been vetoed by the Governor of Massachusetts, so that that distinguished institution has not the power to confer degrees. What has become of the various bills in the last Legislature of New York, for the establishment of Hydropathic, Homœopathic, Eclectic and Patent Medicine Colleges? Most of these had Barnum's name at the head of the Board of Trustees. Since he has "gone up," they must select the next greatest humbug. We suggest Mrs. Fox, the medium.

In Buffalo we have a Spiritual Church, where the new gospel is rapped out piecemeal. It numbers most of our homœopathists as members, our only female physician, and is pretty much made up of similar heretics.

The fight among the Eclectics, at Cincinnati, has been wonderfully amusing. They began by doubting each other's scientific ability, (wherein they were *all* right) then they impeached each other's honesty, (wherein they were all also correct) then they turned out a portion of the faculty (which was wrong, they should have turned them all out); finally the expelled members took forcible possession of the building, thus preventing lectures, and very much disturbing the nerves of the old ladies in the neighborhood, who feared that somebody might be killed (there was no danger if no medicine was taken); the students held meetings, passed indignant resolutions at the baseness of the expelled professors, and at last went home—the very best thing they could do under the circumstances. They should have staid at home in the first place.

The Boston Medical Journal quotes from the Rochester Union an account of a "fish with four legs," obtained in New Mexico. A great flourish of trumpets was made over this fish in the secular papers. A careful reading of the description given makes it evident that it is a variety of the "Proteus," and so far as we can judge it is the common "Syren," of Lake Erie. The point of interest in this animal is not so much its four legs (though those are worthy of remark as forming the most distinct connection between the fish and the reptile) as its branchiæ. The gills are replaced by long waving plumes of blood-vessels, which project an inch from the shoulders, and in which the circulation can be seen with a lens of moderate power. The blood globules are oval and very large—about $\frac{1}{800}$ of an inch in the longer and $\frac{1}{400}$ in the shorter diameter.

